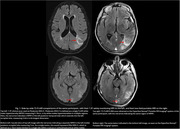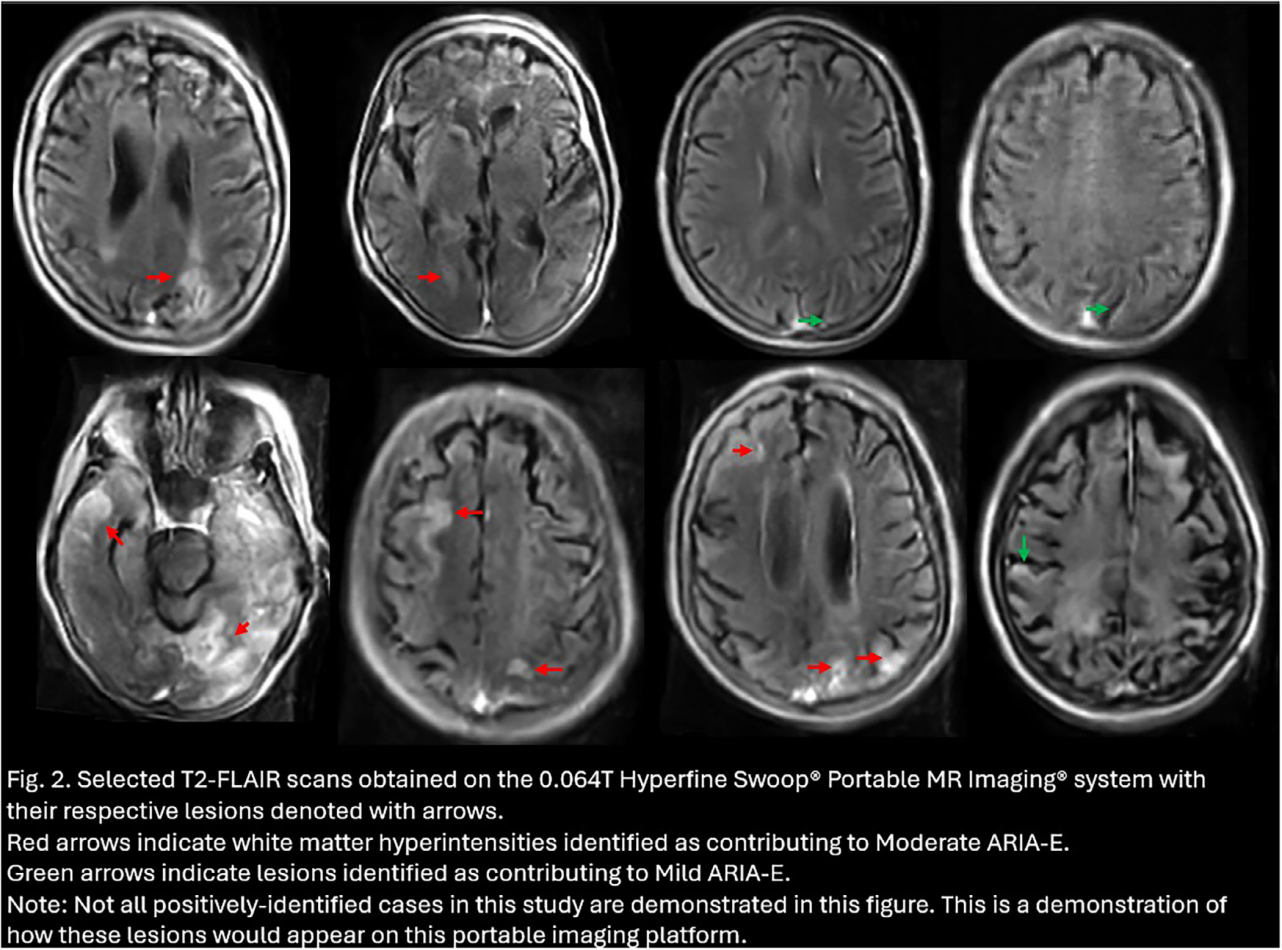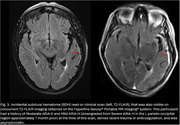# The Use of Portable MRI in the Detection and Monitoring of Amyloid‐Related Imaging Abnormalities

**DOI:** 10.1002/alz70856_104467

**Published:** 2025-12-24

**Authors:** Kavon J Sharifi, Jessica Hu, Hope Shimony, Sydney Nagy, Jude‐Patrick Nnamdi Okafor, Nelly Joseph‐Mathurin, Jessica N Banks, Stephen Jarman, Edmond Knopp, Madeline Paczynski, Alexandra Venuto, Joo Lim, Nupur Ghoshal, B. Joy Snider, Tammie L.S. Benzinger

**Affiliations:** ^1^ Washington University School of Medicine, St. Louis, MO, USA; ^2^ Washington University in St. Louis, St. Louis, MO, USA; ^3^ Washington University School of Medicine in St. Louis, St. Louis, MO, USA; ^4^ Hyperfine, White Plains, NY, USA

## Abstract

**Background:**

Lecanemab is an anti‐beta‐amyloid immunotherapy approved by the FDA in 2023 for Alzheimer's Disease (AD). One known side effect is the development of amyloid‐related imaging abnormalities (ARIA), manifesting as cerebral edema (ARIA‐E), or microhemorrhage with siderosis (ARIA‐H). Appropriate use recommendations for lecanemab recommend brain MRI (clinical scan) at baseline, and approximately 4.5 months, 5.5 months, and 9 months into therapy. The requirement for these scans and the limitations of access to MRI‐capable facilities levies significant burdens on patients, their caregivers, and facilities. Patients who may otherwise benefit have been without this therapy due to lack of MRI access. We aim to demonstrate the viability of an ultra‐low field, portable MRI as an appropriate vehicle for baseline and safety monitoring.

**Method:**

31 patients with AD on lecanemab therapy or off due to known ARIA were recruited for the study. Participants underwent MRI on the low‐field 0.064T Hyperfine Swoop® Portable MR Imaging® system within 1 week of their corresponding clinical screening MRI. Historical data collected from the medical record included age, the reads of baseline and any prior monitoring scans, and ARIA history. The average age of participants was 74.6, and 54.8% were female.

**Result:**

44 scans were obtained on the low‐field MRI that were paired temporally with their clinical counterparts (Figure 1). In total, 14 clinical scans read by a neuroradiologist had at least 1 type and 1 degree of ARIA. Of these, 7 had Mild ARIA‐E, 4 Moderate ARIA‐E, and 8 ARIA‐H (mild to severe). 2 separate, independent neuroradiologists identified all cases of ARIA‐E (mild and moderate, Figure 2) on the low‐field MRI scans. None of the 8 cases of ARIA‐H could be identified on low‐field MRI scans. 1 incidental finding of subdural hematoma (SDH) that was read on a clinical scan was identifiable on its low‐field MRI counterpart (Figure 3).

**Conclusion:**

We found the low‐field MRI to have a 100% sensitivity for both mild and moderate ARIA‐E, but was not sensitive to microhemorrhages. Should this portable MRI modality be further as an adequate surrogate for 1.5/3T clinical scans, it could ease burdens on patients, their caregivers, and hospitals.